# Uptake and predictors of early postnatal follow–up care amongst mother–baby pairs in South Africa: Results from three population–based surveys, 2010–2013

**DOI:** 10.7189/jogh.07.021001

**Published:** 2017-12

**Authors:** Anna Larsen, Mireille Cheyip, Getahun Aynalem, Thu–ha Dinh, Debra Jackson, Nobubelo Ngandu, Witness Chirinda, Mary Mogashoa, Gupreet Kindra, Carl Lombard, Ameena Goga

**Affiliations:** 1 Division of Global HIV/AIDS and Tuberculosis, Center for Global Health, US Centers for Disease Control and Prevention (CDC), Pretoria, South Africa; 2 Division of Global HIV/AIDS and Tuberculosis, Center for Global Health, US Centers for Disease Control and Prevention (CDC), Atlanta, Georgia, USA; 3 Health Systems Research Unit, South African Medical Research Council (HSRU, SAMRC), Pretoria, South Africa; 4 School of Public Health, University of the Western Cape, Cape Town, South Africa; 5 Department of Paediatrics, University of Pretoria, Pretoria, South Africa; 6 United Nations Children’s Fund (UNICEF), New York, New York, USA

## Abstract

**Background:**

Achieving World Health Organization (WHO) recommendations for postnatal care (PNC) within the first few weeks of life is vital to eliminating early mother–to–child transmission of HIV (MTCT) and improving infant health. Almost half of the annual global deaths among children under five occur during the first six weeks of life. This study aims to identify uptake of three PNC visits within the first six weeks of life as recommended by WHO among South African mother–infant pairs, and factors associated with uptake.

**Methods:**

We analyzed data from three facility–based, nationally representative surveys (2010, 2011/12 and 2012/13) primarily designed to determine the effectiveness of the South African program to prevent MTCT. This analysis describes the proportion of infants achieving the WHO recommendation of at least 3 PNC visits. Interviews from 27 699 HIV–negative and HIV–positive mothers of infants aged 4–8 weeks receiving their six week immunization were included in analysis. Data were analyzed using STATA 13.0 and weighted for sample ascertainment and South African live births. We fitted a multivariable logistic regression model to estimate factors associated with early PNC uptake.

**Results:**

Over half (59.6%, 95% confidence interval (CI) = 59.0–60.3) of mother–infant pairs received the recommended three PNC visits during the first 6 weeks; uptake was 63.1% (95% CI = 61.9–64.3) amongst HIV exposed infants and 58.1% (95% CI = 57.3–58.9) amongst HIV unexposed infants. Uptake of early PNC improved significantly with each survey, but varied significantly by province. Multivariable analysis of the pooled data, controlling for survey year, demonstrated that number of antenatal visits (4+ vs <4 Adjusted odds ratio (aOR) = 1.13, 95% CI = 1.04–1.23), timing of initial antenatal visits (≤12 weeks vs >12 weeks, aOR = 1.13, 95% CI = 1.04–1.23), place of delivery (clinic vs hospital aOR = 1.5, 1.3–1.6), and infant HIV exposure (exposed vs unexposed aOR = 1.2, 95% CI = 1.1–1.2) were the key factors associated with receiving recommended PNC visits.

**Conclusions:**

Approximately 40% of neonates did not receive three or more postnatal care visits in the first 6 weeks of life from 2010–2013. To improve uptake of early PNC, early antenatal booking, more frequent antenatal care attendance, and attention to HIV negative women is needed.

Global efforts to improve the health of children under one year of age have succeeded in reducing preventable infant deaths, yet infant mortality remains high in sub–Saharan Africa, especially in settings of high HIV prevalence [1,2]. Neonatal deaths occur predominantly from complications with preterm births, intrapartum–related issues, sepsis, and pneumonia – all requiring medical intervention within the health system if they are to be averted [3]. Most infant deaths occur during the postnatal period from birth to six weeks, making health care interactions important during this critical time [1,3,4]. If mother to child transmission of HIV (MTCT) has not occurred during pregnancy or delivery, it can occur during the early postnatal period, if adherence to maternal triple antiretroviral therapy (ART) is inadequate during breastfeeding and maternal viral load is not suppressed [5]. Interactions with the health system during the early postnatal period can aid in averting these causes of neonatal death and reducing early HIV transmission.

South Africa, as a country with slowly reducing infant mortality and static perinatal mortality, within the context of the greatest HIV epidemic in the world, suffers in the dual and associated burdens of child mortality and HIV [6]. Although infant mortality reduced from 39 per 1000 live births in 2009 to 29 per 1000 live births in 2013, neonatal mortality rates and antenatal HIV prevalence remain stable at 11 per 1000 live births since 2012 [7], and almost one third (29.7%) of pregnant women were living with HIV in 2013 [8]. This ultimately places infant populations at greater risk for HIV acquisition and death [9].

While South Africa demonstrates commitment to adopting and implementing prevention of mother to child transmission (PMTCT) guidelines [10–12], there is a paucity of data on uptake of early postnatal care (PNC). The World Health Organization (WHO) recommends that infants receive at least three PNC visits within the first six weeks of life timed at 3 days, 7–14 days, and 6 weeks to ensure positive health outcomes [4]. The WHO recommends three PNC visits over fewer PNC visits based on evidence regarding the timing and prevalence of causes of infant mortality and morbidity and is further described in the “WHO Recommendations on Postnatal Care of the Mother and Newborn” [4]. It is important to note that the optimum number and timing of PNC visits is the subject of debate, especially in resource limited settings [13]. South Africa has not yet adopted the WHO recommendation and promotes two early PNC visits within 6 days post–delivery and 6 weeks [14]. These visits support infant and child health through delivery of immunizations and dissemination of health messages to mothers such as appropriate feeding practices and recognition of danger signs. No routine home –based PNC visits were part of national policy in South Africa, at the time of this work.

National statistics regarding early PNC focus on attendance to the PNC visit timed before 6 days post–delivery. Estimates from 2014–2015 indicate that about 74.3% of mothers and infants achieved this visit, falling slightly short of the national target of 80% [14].

This paper aims to describe the current status of population–level achievement of at least three PNC facility–based visits during the first six weeks postpartum among infants achieving the 6–week immunization visit in South Africa, and to identify associated factors. This analysis identifies areas of potential intervention to improve progress toward achieving the global WHO recommendation in the interest of optimizing neonatal health.

## METHODS

### Study population

Data for this secondary data analysis were collected through the “Evaluation of the effectiveness of the national Prevention of Mother–to–Child Transmission programme on infant HIV in South Africa” surveys conducted in 2010 (June–December 2010), 2011/12 (August 2011–March 2012), and 2012/13 (October 2012–May 2013). Public sector health facilities were sampled using multi–stage, probability proportional to size methodology, and the study was powered to produce nationally–representative results of MTCT. More detailed information about the survey is available in previous publications [10,15].

For this study, mother/caregiver–infant pairs were enrolled during the infant’s first postpartum immunization visit at six weeks since national coverage of this visit is known to be very high in South Africa [11]. Infants presenting severely ill at the facility were not included in the study, and by nature of the sampling method, infants who died before 6 weeks of age were not included. A total of 30 751 mother/caregiver–infant pairs over the three survey years were eligible for the study and completed interviews.

### Data collection

Trained fieldworkers (nurses) interviewed mothers and caregivers about socio–demographic information, infant health and feeding practices, and postnatal care. Mothers were also interviewed about their HIV status and testing practices, and care received during the antenatal and intrapartum periods. Infant dried blood spot samples (iDBS) were taken at the time of the interview (4–8 weeks postpartum) to detect the presence of maternal HIV antibodies and infant HIV infection. Responses to the question on facility–based PNC were based on maternal recall; however, fieldworkers cross–checked information recalled by the mother with information documented in the infant’s Road to Health booklet. Gestational age was abstracted from the infant Road to Health booklet.

### Data analysis

This particular analysis was restricted to 27 699 mothers (not other caregivers) with available PNC information, regardless of the availability of infant HIV test results (9278 from 2010, 9542 from 2011/12, and 8879 from 2012/13). Data were weighted to adjust for sampling methods, sample ascertainment, and South African live births. We performed frequencies to assess the proportion of infants who achieved at least 3 PNC visits in a facility within the first six weeks post–delivery vs those who had fallen short of the recommended visits. The six–week immunization visit was included as a PNC visit.

We performed frequencies to describe socio–demographic characteristics of the mother–infant pairs, as well as frequencies to examine factors related to antenatal and delivery care. HIV exposure was defined by maternal self–reported HIV status during pregnancy and labor. To determine whether variables predicting uptake of early PNC were different by HIV exposure status, we tested the associations between each variable of interest and the PNC outcome variable separately among HIV exposed infants and among HIV unexposed infants. When it was established that the same associations were seen regardless of HIV exposure status, we pooled these data. Justification was also established to pool the data from all survey years when significant changes were not seen among independent variables over time.

To estimate uptake of early PNC as an odds ratio adjusted for all covariates, we fitted a multivariable logistic regression model using purposeful selection of variables [12]. Parameters were initially included in the multivariable model if they had a Wald test result that was significant at 25% in bivariate logistic regression. Prior studies indicate that traditional cut–off levels for significance, such as the commonly used 5% significance level, fail to identify important variables [16,17]. Variables were then eliminated from the model if they were no longer significant at 10% in the multivariable model and if their removal did not change any parameter by more than 15%. Variables not significant in bivariate regression were then placed in the model and retained if they became significant when added to the multivariable model or if they changed any parameter by more than 15% [12]. All analyses were performed using STATA 13.

### Ethical consideration

The protocol for the cross–sectional survey was approved by Human Subjects Division at the United States Centers for Disease Control and Prevention within the Center for Global Health and the institutional review board of the South African Medical Research Council. Mothers provided written informed consent prior to the onset of the interview.

## RESULTS

### Population characteristics

Within the population included for this analysis, one third (30.0%, 95% CI = 29.4–30.6) of infants were HIV exposed based on maternal self–reported HIV status (**Table 1**). Most infants were 6 weeks old (79.9%, 95% CI = 79.3–80.4) at the time of the interview, while smaller proportions were 4–5 weeks old or 7–8 weeks old. Almost all infants were black (92.8, 95% CI = 92.5–93.1).

**Table 1:** Characteristics of study population, overall and by year (N = 27 699)

	Total N = 27 699		Survey year 2010, n = 9278	Survey year 2011, n = 9542	Survey year 2012, n = 8879
	**Unweighted N (%)**	**Weighted %, (95% CI)**	**Weighted % (95% CI)**	**Weighted % (95% CI)**	**Weighted % (95% CI)**
**Infant HIV exposure**					
**Mother's self–reported HIV status (proxy for HIV exposure):**					
HIV unexposed	19 035 (68.72)	66.80 (66.16–67.43)	67.73 (66.59–68.85)	65.90 (64.81–66.97)	66.79 (65.70–67.86)
HIV exposed	7693 (27.77)	30.00 (29.39–30.63)	29.79 (28.69–30.92)	28.88 (27.84–29.94)	30.00 (29.39–30.63)
No response (NR)	895 (3.23)	2.95 (2.74–3.17)	2.13 (1.83–2.49)	4.96 (4.51–5.45)	2.95 (2.74–3.17)
Chose not to answer	76 (0.27)	0.25 (0.20–0.32)	0.34 (0.24–0.50)	0.26 (0.18–0.39)	0.25 (0.20–32.02)
**Maternal characteristics:**					
**Province**					
Gauteng	4673 (9.24)	26.83 (26.19–27.47)	29.56 (28.37–30.79)	27.09 (26.02–28.20)	23.88 (22.88–24.90)
Eastern Cape	2560 (9.24)	9.63 (9.27–10.01)	8.48 (7.81–9.19)	9.41 (8.85–9.99)	10.99 (10.33–11.68)
Free State	2947 (10.64)	4.96 (4.78–5.16)	4.96 (4.66–5.28)	4.77 (4.45–5.10)	5.17 (4.83–5.52)
Kwa–Zulu Natal	3356 (12.12)	21.43 (20.81–22.07)	21.17 (20.63–22.82)	21.46 (20.40–22.57)	21.13 (20.05–5.53)
Limpopo	3077 (11.11)	10.81 (10.44–11.19)	9.99 (9.37–10.64)	10.64 (10.01–11.31)	11.78 (11.14–12.44)
Mpumalanga	3211 (11.59)	7.79 (7.52–8.06)	7.64 (7.21–8.09)	7.88 (7.43–8.35)	7.84 (7.33–8.37)
Northern Cape	1227 (4.43)	2.04 (1.93–2.16)	1.69 (1.51–1.89)	2.21 (2.02–2.42)	2.22 (2.01–2.44)
North West	2949 (10.65)	7.46 (7.19–7.74)	7.44 (7.01–7.89)	7.19 (6.76–7.65)	7.75 (7.24–8.30)
Western Cape	3699 (13.35)	9.05 (8.76–9.35)	8.54 (8.06–9.04)	9.35 (8.86–9.87)	9.25 (8.74–9.80)
**Mean age (standard deviation)**	26.14 (6.29)	26.11 (6.28)	25.95 (6.18)	26.09 (6.37)	26.29 (6.30)
**Age category:**					
≤19	4188 (15.12)	15.15 (14.67–15.63)	15.47 (14.63–16.35)	15.92 (15.12–16.77)	14.05 (13.26–14.87)
20–24	8395 (30.31)	30.52 (29.91–31.13)	30.45 (29.36–31.57)	30.18 (29.15–31.22)	30.91 (29.87–31.97)
25–34	11 763 (42.47)	42.29 (41.64–42.95)	42.54 (41.36–43.73)	41.42 (40.31–42.53)	42.93 (41.82–44.05)
≥35	3282 (11.85)	11.75 (11.33–12.18)	10.63 (9.92–11.38)	12.48 (11.75–13.25)	12.11 (11.40–12.86)
NR	71 (0.26)	0.30 (0.23–0.39)	0.91 (0.69–1.19)	0 (0.00)	0 (0.00)
**Level of education:**					
Grades 1–7	3996 (14.42)	13.61 (13.17–14.06)	14.54 (13.74–15.38)	12.95 (12.23–13.71)	13.37 (12.63–14.14)
Grades 8–12	21 795 (78.69)	79.27 (78.73–79.80)	78.14 (77.15–79.09)	79.85 (78.93–80.73)	79.80 (78.89–80.68)
Completed tertiary/ technical/ university	1459 (5.27)	5.60 (5.30–5.92)	5.42 (4.89–5.99)	5.75 (5.24–6.31)	5.65 (5.15–6.18)
None/NA	449 (1.62)	1.52 (1.37–1.68)	1.91 (1.63–2.25)	1.46 (1.22–1.74)	1.19 (0.97–1.46)
**Marital status:**					
Single/widowed/divorced/separated	20 393 (73.62)	75.46 (74.90–76.01)	75.36 (74.33–76.35)	75.07 (74.11–76.01)	75.96 (75.01–76.88)
Married/co–habiting	7306 (26.38)	24.54 (23.99–25.10)	24.64 (23.65–25.67)	24.93 (23.99–25.89)	24.04 (23.12–24.99)
**Parity:**					
Multiparous	16 868 (60.90)	61.08 (60.43–61.72)	59.50 (58.32–60.66)	61.43 (60.33–62.52)	62.28 (61.17–63.37)
Primiparous	10 831 (39.10)	38.92 (38.28–39.57)	40.50 (39.34–41.68)	38.57 (37.48–39.67)	37.72 (36.63–38.83)
**Correct identification of all MTCT modes:**					
Yes	15 166 (54.75)	55.86 (55.20–56.52)	46.83 (45.64–48.03)	61.39 (60.30–62.47)	59.16 (58.05–60.26)
No	12 533 (45.25)	44.14 (43.48–44.80)	53.17 (51.97–54.36)	38.61 (37.53–39.70)	44.14 (43.48–44.80)
**Infant characteristics:**					
**Gender:**					
Male	13 926 (50.28)	50.34 (49.67–51.00)	50.26 (49.07–51.46)	50.11 (48.98–51.24)	50.64 (49.51–51.77)
Female	13 773 (49.72)	49.66 (49.00–50.33)	49.74 (48.54–50.93)	49.89 (48.76–51.04)	49.36 (48.23–50.49)
**Age:**					
4–5 weeks	1959 (7.07)	6.28 (5.98–6.59)	7.83 (7.26–8.45)	7.18 (6.64–7.76)	3.85 (3.44–4.29)
6 weeks	21 753 (78.53)	79.86 (79.33–80.38)	73.64 (72.60–74.65)	80.49 (79.60–81.36)	85.32 (84.51–86.09)
7–8 weeks	3987 (14.39)	13.87 (13.42–14.32)	18.53 (17.64–19.45)	12.33 (11.61–12.08)	10.84 (10.16–11.55)
**Population group:**					
Black	24 911 (89.93)	92.79 (92.49–93.07)	92.99 (92.46 – 93.48)	92.33 (91.81 – 92.82)	93.04 (92.54 – 93.52)
White	135 (0.49)	0.47 (0.39 - 0.57)	59.32 (43.11 - 81.57)	0.46 (0.34–0.64)	0.35 (0.25–0.51)
Coloured	2541 (9.17)	6.27 (6.01–6.53)	5.861 (5.43–6.32)	6.69 (6.26–7.16)	6.24 (5.79–6.71)
Indian	73 (0.26)	0.36 (0.28–0.46)	0.43 (0.28–0.65)	0.40 (0.27–0.61)	0.25 (0.16–0.40)
Other	39 (0.14)	0.12 (0.08–0.16)	0.13 (0.08–0.22)	0.11 (0.05–0.21)	0.11 (0.06–0.20)
**Gestational age from Road to Health Booklet:**					
≥37 weeks	18 843 (68.03)	69.05 (68.44–69.65)	82.03 (81.12–82.90)	62.28 (61.19–63.35)	63.11 (62.02–64.18)
<37 weeks	3646 (13.16)	12.62 (12.19–13.06)	17.97 (17.10–18.88)	10.04 (9.40–10.72)	9.97 (9.32–10.67)
NR	5210 (18.18)	18.33 (17.84–18.83)	0 (0.00)	27.68 (26.70–28.69)	26.92 (25.95–27.91)
**Hospitalized within first 6 weeks of life:**					
No	25 708 (92.81)	92.88 (92.53–93.22)	91.04 (91.34–91.69)	91.67 (91.01–92.28)	95.90 (95.41–96.35)
Yes	1966 (7.10)	7.06 (6.72–7.41)	8.88 (8.23–9.58)	8.28 (7.67–8.93)	4.04 (3.60–4.53)
NR	25 (0.09)	.06 (0.04–0.09)	0.08 (0.04–0.15)	0.05 (0.03–0.11)	0.06 (0.03–0.13)
**ANC and delivery characteristics:**					
**Received support from community health worker:**					
No	8953 (32.32)	31.51 (30.90–32.13)	38.75 (37.59–39.93)	26.35 (25.40–27.32)	29.59 (28.59–30.62)
Yes	14 419 (52.06)	51.39 (50.72–52.05)	61.25 (60.07–62.41)	48.30 (47.17–49.42)	44.82 (43.69–45.95)
NR	4327 (15.62)	17.1 (16.61–17.61)	0 (0.00)	25.36 (24.36–26.37)	25.59 (24.61–26.59)
**ANC visits:**					
0–3 visits	4382 (15.82)	16.43 (15.94–16.94)	19.14 (18.20–20.13)	15.85 (15.04–16.68)	14.37 (13.60–15.17)
4–5 visits	8212 (29.65)	30.66 (30.05–31.28)	28.39 (27.13–29.49)	31.94 (30.90–33.01)	31.61 (30.57–32.67)
≥6 visits	6519 (23.54)	22.77 (22.22–23.33)	19.91 (18.99–20.86)	23.06 (22.14–24.02)	25.27 (24.29–26.28)
NR	8586 (31.00)	30.13 (29.53–30.74)	32.56 (31.47–33.67)	29.15 (28.13–30.19)	28.75 (27.73–29.78)
**Timeliness of first ANC visit:**					
≤12 weeks	7695 (27.78)	26.20 (25.63–26.78)	23.46 (22.49–24.45)	25.19 (24.24–26.17)	29.92 (28.89–30.95)
13–20 weeks	10 061 (36.32)	37.25 (36.61–37.90)	33.60 (32.48–34.74)	36.90 (35.82–38.00)	41.18 (40.07–42.30)
≥21 weeks	6536 (23.60)	24.55 (23.98–25.13)	28.95 (27.86–30.06)	23.44 (22.49–24.42)	21.35 (20.44–22.29)
NR	3407 (12.30)	11.99 (11.57–12.43)	13.99 (13.19–14.84)	14.46 (13.69–15.27)	7.55 (6.99–8.15)
**Delivery location:**					
Hospital	21 466 (77.50)	78.14 (77.59–78.67)	77.88 (76.88–78.85)	78.63 (77.70–79.53)	77.89 (76.94–78.80)
Clinic	4724 (17.05)	16.61 (16.13–17.10)	16.39 (15.53–17.29)	15.68 (14.88–16.50)	17.76 (16.92–18.63)
Home/other	1282 (4.63)	4.48 (4.21–4.76)	5.73 (5.20–6.30)	4.28 (3.85–4.75)	3.46 (3.06–3.90)
NR	227 (0.82)	0.78 (0.68–0.89)	0 (0.00)	1.42 (1.19–1.68)	0.90 (0.71–1.14)
**Delivery method:**					
Vaginal delivery	21 424 (77.35)	76.83 (76.26–77.39)	78.51 (77.51–79.48)	75.48 (74.48–76.45)	76.55 (75.57–77.50)
Caesarean section	5957 (21.51)	22.17 (21.62–22.73)	21.42 (20.46–22.42)	21.75 920.81–22.71)	23.33 (22.38–24.31)
Do not know	38 (0.14)	0.12 (0.08–0.17)	0.07 (0.04–0.13)	0.18 (0.10–0.30)	0.12 (0.07–0.20)
NR	280 (1.01)	0.87 (0.76–1.00)	0 (0.00)	2.60 (2.27–2.97)	0 (0.00)

NR – not recorded

The mean age of mothers was 26.1 years (standard deviation: 6.3) and the majority of them were single (75.5%, 95% CI = 74.9–76.1). Most women reported that their highest level of education attended was grades 8–12 (79.3%, 95% CI = 78.7–79.8). Over half of the women were multiparous (61.1%, 95% CI = 60.4–61.7). Over half of the women (55.9%, 95% CI = 55.2–56.5) were able to correctly identify all modes of MTCT.

### Uptake of early PNC

About 60% (59.6%, 95% CI = 59.0–60.3) of those included in the survey achieved at least 3 PNC visits in the first 6 weeks of life, with a median of 3 PNC visits per infant (**Table 2**). Approximately 46.0% (95% CI = 44.8–47.2) of respondents from the 2010 survey, 58.2% (95% CI = 57.1–59.0) from the 2011/12 survey, and 74.4% (95% CI = 73.4–75.3) of respondents from the 2012/13 survey year achieved the three WHO recommended PNC visits. The province with the highest proportion of infants receiving at least 3 PNC was Free State (85.1%, 95% CI = 83.7–86.4). The province with the lowest proportion of infants receiving at least 3 PNC was Northern Cape (2.04%, 95% CI = 1.93–2.16). Notably, over 94% (94.36, 95% CI = 92.48–96.30) of infants at the 6–week immunization visit had achieved at least 2 early PNC visits (the current guideline in South Africa).

**Table 2:** Pooled analysis of uptake of ≥3 postnatal care visits in the first six weeks post–delivery

	Total N = 27 699	
**Postnatal care utilization**	**Unweighted**	**Weighted**
	**N (%)**	**% (95% CI)**
**Percent of participants achieving ≥3 postnatal visits, by province**	**≥3 PNC**	**≥3 PNC**
**South Africa (National)**	**17 351 (62.64)**	**59.61 (58.95–60.26)**
Gauteng	2238 (47.89)	46.89 (45.43–48.35)
Eastern Cape	1115 (43.55)	43.94 (41.94–45.96)
Free State	2518 (85.44)	85.08 (83.70–86.37)
Kwa–Zulu Natal	2190 (65.26)	66.36 (64.70–67.98)
Limpopo	1776 (57.72)	56.81 (55.02–58.59)
Mpumalanga	2148 (66.90)	67.97 (66.31–69.59)
Northern Cape	768 (62.90)	62.92 960.16–65.60)
North West	2330 (79.01)	81.13 (79.72–82.47)
Western Cape	2268 (61.31)	61.67 (60.08–63.24)
**Percent of participants achieving ≥3 postnatal visits, by HIV exposure**		
HIV unexposed	11 661 (61.25)	58.06 (57.26–58.85)
HIV exposed	5076 (65.95)	63.09 (61.86–64.30)
Unknown/Chose not to respond	349 (36.24)	59.30 (55.74–62.77)
**Percent of participants achieving ≥3 postnatal visits, by survey year**		
2010	5040 (54.32)	46.02 (44.84–47.20)
2011/12	5784 (60.62)	58.19 (57.07–59.30)
2012/13	6527 (73.51)	74.35 (73.37–75.31)
**Number of early postnatal care visits (National)**		
0 visits	0 (0.00)	0 (0.00)
1 visit	1230 (4.44)	5.63 (5.31–5.96)
2 visits	9118 (32.92)	34.76 (34.13–35.40)
3 visits	13 511 (48.78)	46.95 (46.29–47.62)
4 visits	2678 (9.67)	8.98 (8.61–9.36)
5+ visits	1162 (4.20)	3.67 (3.45–3.92)
**Average postnatal visits**		
Median (Quartile 1, Quartile 3)	3 (2, 3)	3 (2, 3)

PNC – postnatal care

### Factors associated with early PNC uptake

A higher proportion of infants who were HIV exposed achieved 3 PNC (63.1%, 95% CI = 61.9–64.3), compared to HIV unexposed infants (58.1%, 95% CI = 57.3–58.9) (**Table 3**). This relationship persisted (aOR = 1.2, 95% CI = 1.1–1.2) when adjusted for all other covariates (**Table 4****)**.

**Table 3:** Factors associated with uptake of ≥3 early PNC visits in the first 6 weeks post–delivery

	Unweighted proportion achieving ≥3 PNC by covariate level (x/n)	Weighted percentage achieving ≥3 PNC by covariate level, % (95% CI)	Weighted, Unadjusted OR, (≥3 PNC visits vs <3 PNC visits)	95% CI
**Infant HIV exposure:**				
**Mother's self–reported HIV status:**				
HIV unexposed	11 661/19 039	58.06 (57.26–58.85)	1.00	
HIV exposed	5076/7697	63.09 (61.86–64.30)	1.23	1.16–1.31
NR/Chose not to respond*	614/963	59.30 (55.74–62.77)	1.05	0.91–1.22
**Maternal characteristics:**				
**Province:**				
Gauteng	2238/4673	46.89 (45.43–48.35)	1.00	
Eastern Cape	1115/2560	43.94 (41.94–45.96)	0.89	0.80–0.98
Free State	2518/2947	85.08 (83.70–86.37)	6.46	5.73–7.29
Kwa–Zulu Natal	2190/3356	66.36 (64.70–67.98)	2.23	2.03–2.45
Limpopo	1776/3077	56.81 (55.02–58.59)	1.49	1.36–1.64
Mpumalanga	2148/3211	67.97 (66.31–69.59)	2.40	2.19–2.64
Northern Cape	768/1227	81.13 (79.72–82.47)	1.92	1.69–2.19
North West	2330/2949	61.67 (60.08–63.24)	4.87	4.38–5.42
Western Cape	2268/3699	59.61 (58.95–60.26)	1.82	1.67–1.99
**Age:**				
≤19	2575/4188	58.74 (57.03–60.43)	1.00	
20–24	5289/8395	59.82 (58.62–61.01)	1.05	0.96–1.14
25–34	7421/11 763	60.16 959.15–61.16)	1.06	0.98–1.15
≥35	2031/3282	58.69 (56.77–60.58)	0.99	0.90–1.11
NR	35/ 71	40.00 (27.94–53.41)	0.47	0.27–0.81
**Level of education:**				
Grades 1–7	2556/3996	60.44 (58.69–62.16)	1.00	
Grades 8–12	13 783/21 795	60.45 (59.71–61.18)	1.00	0.92–1.08
Completed tertiary/ technical/ university	743/1459	46.24 943.44–49.05)	0.56	0.49–0.64
None/NR	269/ 449	57.68 (52.44–62.75)	0.89	0.71–1.12
**Marital status:**				
Single/widowed/divorced/separated	12873/20 393	60.56 (59.80–61.32)	1.00	
Married/co–habiting	4478/7306	56.68 (55.38–57.96)	0.85	0.80–0.91
**Parity:**				
Multiparous	10 635/16 868	60.27 (59.43–61.10)	1.00	
Primiparous	6716/10 831	58.57 (57.51–59.63)	0.93	0.88–0.99
**Correct identification of all MTCT modes:**				
Yes	9656/15 166	60.94 (60.06–61.82)	1.00	
No	7695/12 533	57.92 (56.93–58.90)	0.88	0.84–0.93
**Infant characteristics:**				
**Gender:**				
Male	8723/13 926	59.38 (58.45–60.30)	1.00	
Female	8628/13 773	59.84 (58.91–60.77)	1.02	097–1.08
**Age:**				
4–5 weeks	1202/1959	58.38 (55.85–60.86)	0.93	0.83–1.03
6 weeks	13 733/21 753	60.25 (59.51–60.99)	1.00	
7–8 weeks	2376/3987	56.44 (54.68–58.18)	0.85	0.79–0.92
**Population group:**				
Black	15567/24 911	59.51 (58.82–60.20)	1.00	
Non–black	1784/2788	60.83 (58.76–62.85)	1.06	0.96–1.16
**Gestational age from the Road to Health Booklet:**				
≥37 weeks	11 433/18 843	57.20 (56.39–58.00)	1.00	
<37 weeks	2249/3646	57.81 (55.97–59.63)	1.03	0.94–1.11
NR	3669/5210	69.92 (68.54–71.26)	1.74	1.62–1.87
**Hospitalized within first 6 weeks of life:**				
No	15 985/25 708	59.04 (58.36–59.72)	1.00	
Yes	1351/1966	67.03 (64.59–69.38)	1.41	1.26–1.58
NR	15/ 25	58.08 (37.38–76.28)	0.96	0.41–2.23
**ANC and delivery characteristics:**				
**Received support from community health worker:**				
No	5713/8953	62.24 (61.10–63.38)	1.00	
Yes	8837/14 419	56.08 (55.15–57.01)	0.77	0.73–0.82
NR	2801/4327	65.33 (63.80–66.84)	1.14	1.05–1.24
**ANC visits:**				
<4 visits	2571/4382	55.27 (53.61–56.92)	1.00	
≥4 visits	14 780/23 317	59.57 (58.37–60.76)	1.24	1.15–1.33
**Timeliness of first ANC visit:**				
>12 weeks	10 404/16 597	59.83 (58.98–60.67)	1.00	
≤12 weeks	5116/7695	64.69 (63.46–65.89)	1.23	1.15–1.31
NR	1831/3407	47.37 (45.49–49.26)	0.60	0.56–0.66
**Delivery location:**				
Hospital	13 047/21 466	57.53 (56.78–58.28)	1.00	
Clinic	3326/4724	68.86 (67.35–70.34)	1.63	1.51–1.76
Home/other/NR	978/1509	61.24 (58.37–64.03)	1.17	1.03–1.32
**Delivery method:**				
Vaginal delivery	13 766/21 424	61.25 (60.51–61.99)	1.00	
Caesarean section	3364/5597	53.79 (52.36–55.22)	0.74	0.69–0.79
DK/NR	221/ 318	62.02 (55.59–68.06)	1.03	0.79–1.35
**Survey year:**				
2010	5040/9278	46.02 (44.84–47.20)	1.00	
2011/12	5784/9542	58.19 (57.07–59.30)	1.63	1.53–1.74
2012/13	6527/8879	74.35 (73.37–75.31)	3.40	3.17–3.65

CI – confidence interval, PNC – postnatal care, NR – no response, ANC – antenatal care

*Categories of “Don't know” “No response” “None” and/or “Chose not to answer” were combined in cases where one or both categories were <1%. See **Table1** for full descriptive report.

**Table 4:** Multivariable logistic regression of factors associated with uptake of ≥3 early PNC visits in the first 6 weeks post–delivery*

Parameter	Variable	≥3 early PNC visits vs <3 PNC visits	
		**Weighted Adjusted OR**	**95% CI**
**Infant HIV exposure:**			
**Mother's self–reported HIV status (proxy for HIV exposure)**	HIV exposed vs HIV unexposed	1.16	1.10–1.23
**Maternal characteristics:**			
**Age (years):**			
	20–24 vs ≤19	1.06	0.97–1.17
	25–34 vs ≤19	1.11	1.01–1.21
	≥35 vs ≤19	1.05	0.93–1.19
**Level of education**	Grades 8–12 vs Grades 1–7	1.05	0.96–1.14
	Completed tertiary/ technical/ university vs Grades 1–7	0.67	0.58–0.77
	None/NA vs Grades 1–7	0.92	0.72–1.17
**Marital status**	Married/co–habiting vs Single/widowed/divorced/separated	0.94	0.88–1.01
**Correct identification of all MTCT modes**	No vs Yes	0.90	0.85–0.96
**Province**	Eastern Cape vs Gauteng	0.76	0.68–0.85
	Free State vs Gauteng	6.60	5.79–7.52
	Kwa–Zulu Natal vs Gauteng	2.15	1.94–2.37
	Limpopo vs Gauteng	1.27	1.15–1.41
	Mpumalanga vs Gauteng	2.42	2.18–2.68
	Northern Cape vs Gauteng	1.55	1.33–1.80
	North West vs Gauteng	4.22	3.78–4.72
	Western Cape vs Gauteng	1.71	1.52–1.91
**Infant characteristics:**			
Population group	Non–black vs Black	1.21	1.08–1.37
Gestational age	<37 weeks vs ≥37 weeks	1.22	1.11–1.34
Hospitalized within first 6 weeks of life	Yes vs No	1.62	1.44–1.83
**ANC and delivery characteristics:**			
**Received support from community health worker (CHW)**	CHW support vs No CHW support	0.87	0.81–0.93
**ANC visits**	≥4 visits vs <4 visits	1.13	1.04–1.23
**Timeliness of first ANC visit**	≤12 weeks vs >12 weeks	1.13	1.04–1.23
**Delivery location**	Clinic vs Hospital	1.45	1.33–1.58
	Home/other vs Hospital	1.14	0.99–1.32
**Delivery method**	Caesarean vs Vaginal	0.74	0.68–0.79
**Survey year**	2011/12 vs 2010	1.61	1.49–1.73
	2012/13 vs 2010	3.49	3.21–3.79

CI – confidence interval, PNC – postnatal care, OR – odds ratio

*All “Don't know” or “No answer” categories were not included since they are not interpretable.

Province of residence was highly associated with uptake of WHO recommended early PNC. Infants residing in all provinces (except Eastern Cape) were at least 30% more likely to receive 3 or more early PNC visits compared to infants in Gauteng Province, with those in Free State Province almost seven times more likely to receive recommended PNC (aOR = 6.6, 95% CI = 5.8–7.5) compared with Gauteng Province residents.

Mothers who delivered in a clinic vs a hospital, who achieved at least four ANC visits, or who booked an ANC appointment before 12 weeks gestation were far more likely to achieve at least 3 recommended early PNC visits (aOR = 1.5, 95% CI = 1.3–1.6, ; aOR = 1.1, 95% CI = 1.04–1.2, and aOR = 1.13, 95% CI = 1.04–1.23, respectively). However, those who received support during pregnancy and delivery from a community health worker (CHW) were less likely than those who did not receive this support to have at least 3 early PNC visits (aOR = 0.9, 95% CI = 0.8–0.9). Those who delivered via Caesarean section had lower achievement of early PNC visits than those with vaginal delivery (aOR = 0.7, 95% CI = 0.7–0.8).

Infants who required hospitalization during the first six weeks of life were more likely than those who were not hospitalized to have 3 early PNC visits (aOR = 1.6, 95% CI = 1.4–1.8). Similarly, those born prematurely (before 37 weeks gestational age) were more likely to achieve the recommended 3 PNC visits than those born at full term (aOR = 1.6, 95% CI = 1.4–1.8). Infants classified as Non–Black (Coloured, White, Indian, or other) were more likely to achieve 3 early PNC visits compared with infants classified as Black (aOR = 1.2, 95% CI = 1.1–1.4).

## DISCUSSION

The WHO recommends that all mother–infant pairs should receive three postnatal care visits during the first 6 weeks of life. Our analysis of data from three nationally representative population–based surveys demonstrated that approximately 60% of infants achieving the 6–week immunization visit access the WHO recommended number of postnatal care visits in the first six weeks of life. Access to early postnatal care visits significantly increased between 2010 and 2012.

This study was the first of its kind to assess achievement of the WHO recommendation for early PNC at a national level in South Africa, and to track changes in access to early PNC over time (2010–2013).

Our findings are consistent with the levels of early PNC uptake documented by the 2014–15 District Health Barometer which, using routine data, demonstrated 74.3% achievement of the first PNC visit within the first 6 days post–delivery [14].

Although approximately 40% of infants did not receive the required number of early PNC visits, our finding that the majority (59.6%) of infants received three or more PNC visits indicates a much higher early PNC coverage in South Africa, an upper middle income country, compared with rural Tanzania and Ghana, low income and lower middle income countries respectively [18]. These countries reported that fewer than 10% of women achieved three or more PNC [19,20]. Identification of factors associated with achieving this recommendation is necessary to inform programmatic efforts that improve coverage in South Africa and other sub–Saharan African countries.

### Factors associated with early PNC uptake

Achievement of recommended interactions with the health system along the continuum of care has been shown to arise from a combination of health system, community, household, and individual–level determinants [21–26]. This study focused on individual–level predictors such as maternal socio–demographic factors, infant HIV exposure, infant socio–demographic factors, and health seeking behavior during pregnancy and delivery.

#### Province of residence

Adherence to WHO recommended early PNC was highly influenced by province of residence, with those residing in Gauteng Province less likely than those in almost every other province to achieve at least 3 early PNC visits. Eastern Cape Province was the only province that had lower achievement of at least 3 PNC visits than Gauteng Province. Populations in Gauteng and Eastern Cape Provinces are highly mobile and thus may demonstrate reduced consistency in attendance to health visits [14,27,28]. Populations with high mobility have been shown to have reduced health seeking behavior and access to care globally [29]. Such mobility may also contribute to mothers seeking PNC in provinces other than their province of residence, which was not captured in the questionnaire. Differences in uptake of early PNC observed between provinces are likely explained by a complex network of differences in relative strength of the provincial health systems, messaging surrounding the importance of PNC to infant health, the general status of health, and geography.

#### HIV exposure status

An infant’s exposure to HIV was found to be predictive of meeting recommended early PNC visits, with HIV exposed infants being more likely to receive at least three PNC visits than HIV unexposed infants. Since the early 2000s, South Africa has aggressively adopted each updated WHO recommendation for PMTCT, and has update postnatal care guidelines [30–35]. Programmatic PMTCT messages emphasize the importance of three or more early PNC visits for maternal and infant health [5]. The targeted nature of these messages to HIV positive mothers within PMTCT programming along the continuum of care likely contributes to the differential in PNC uptake among HIV exposed and unexposed infants. The care provided in PMTCT programs likely incentivizes HIV positive mothers to seek PNC at a higher rate than those perceiving themselves as HIV negative. On a population level this is concerning as HIV negative women comprise the majority of the population. Similarly, HIV positive women have been shown to be more likely to practice safer infant feeding than HIV negative mothers [36].

#### Continuum of care

Our findings show that a woman’s compliance with antenatal recommendations were predictive of early PNC uptake for her infant, as seen in similar studies [19,20]. Uptake of ANC within the first 12 weeks of pregnancy and achievement of at least four ANC visits constitute the recommended timing and frequency of interaction with the health system during pregnancy [37]. This finding indicates that promotion of vital health visits taking place earlier in the continuum of care also work to encourage visits later in the continuum of care.

Receipt of support by a CHW during pregnancy, delivery, and/or postnatal care had the opposite effect on uptake of early PNC. CHW have been increasingly adopted in South Africa as a method to improve access to important health messages and services through lower–level cadres of health workers who are more mobile within the community [38,39]. Results from a randomized control trial in Western Cape, South Africa indicated improved outcomes for mothers and infants from home visits by CHW [40]. By improving health understanding among mothers and by referring infants to higher levels of care when necessary, CHWs may reduce the need for all mother–infant pairs to seek early PNC in a clinic setting [41]. We did not collect Information in this national survey regarding frequency and timing of postnatal care provided by CHW. The relationship seen by this analysis indicating lower achievement of 3 early, facility–based PNC visits among those who had received antenatal or delivery care by a CHW could be explained by mothers receiving care from CHW in the early postnatal period instead of in facilities. Replacement of facility visits by CHW visits and the potential associated differences or similarities in health outcomes should be assessed separately to understand whether CHW are having the intended effect of reducing health system burdens and maintaining or improving maternal and child health in South Africa.

Women who delivered in a clinic were more likely than those who delivered in a hospital or outside of a facility to achieve three early PNC visits. While any facility delivery (hospital or clinic) has been observed in similar studies as a predictor of PNC [19,20] compared to non–facility delivery, our findings distinguished that clinic delivery led to higher likelihood of three early PNC visits than hospital delivery. While ANC services are provided at most clinics, only hospitals and maternity outpatient units (MOU) perform deliveries [31]. It is likely that many of the women who indicated delivering in a “clinic” in the current study actually delivered in a MOU and received ANC services there. These women likely returned to the MOU for PNC services. The women who delivered in a hospital were referred to the hospital by the clinic where they received ANC. It is known that 20% of ANC patients are lost in the referral process to delivery care [42], indicating that inconsistent receipt of care along the continuum may influence failure to achieve recommended early PNC. Additional costs incurred from hospital delivery, such as transport costs if the facility is far, may also deter continuation of care with early PNC visits [24-26,31].

Infants delivered through Caesarean section vs vaginal delivery were less likely to achieve 3 early PNC visits. This is surprising due to the high risk nature of Caesarean delivery, yet may be explained by longer duration in the hospital reducing the need for early PNC visits. This further supports the finding that hospital delivery was associated with lower uptake of early PNC visits.

#### Correct identification of MTCT modes and health seeking behavior

The positive relationship between health knowledge and health seeking behavior by mothers has been shown in multiple studies [43,44] and was supported by our findings. Mothers unable to identify all three modes of MTCT (pregnancy, delivery, and breastfeeding), were less likely to comply with recommendations for early PNC than mothers who correctly identified all MTCT modalities. PMTCT messaging which empowers mothers with knowledge of MTCT modes and preventive behaviors appears to be succeeding in encouraging interaction with the health system during the early postnatal period.

Contrarily, mothers with a high level of education (tertiary/technical, or university education) were less likely than those with a low achievement of education (grade 1–7) to seek at least 3 PNC visits during the first 6 weeks. Studies examining relationships between knowledge, risk perception, and health behaviors indicate that low understanding of a disease may influence perception of high risk and, in turn, perception of high risk motivates health seeking behavior [45–47]. This could imply that mothers with low levels of education, who have low confidence in their own knowledge of health, are more compliant with health professional recommendations, while those with higher education feel confident in their understanding of health and seek less health care. Further, mothers with higher education may be employed at higher levels than those with lower education, leading to time conflicts that reduce health seeking.

#### Infant and maternal demographic characteristics

Premature infants are at higher risk for adverse health in early infancy and thus are recommended to receive more than three early PNC visits, so this finding that premature infants achieve at least 3 visits at a higher frequency than full–term infants is consistent with expectations [4]. Our finding concerning racial differential in PNC health seeking is consistent with previous findings that children of non–black ethnicity achieve higher levels of health care than black children [48,49]. Health messaging and programming should be equitable across ethnic groups and should stress the importance of early PNC. Health care access, finances, time available, and employment status influence health care seeking and likely contribute to this racial differential.

Women aged 25–34 were more likely than adolescent women (19 and younger) to achieve recommended PNC. Related literature is inconclusive about the relationship between maternal age and health seeking behavior, however our finding is reflective of observations from studies in similar settings indicating that younger and less experienced women are less likely to adhere to recommended health care [50,51]. Attendance to at least three early PNC visits should be emphasized to all mothers regardless of age and perceived experience.

Respondents from the 2011/12 and 2012/13 survey years were much more likely to succeed in reaching the recommendation for early PNC than the 2010 survey year. This suggests a pattern of improvement in compliance with WHO recommended early PNC over time, likely due to the influence of non–governmental organizations partnering with the South African government to implement the PMTCT program. From 2008 to 2010 South Africa revised national PMTCT guidelines, adding specification of a postnatal visit within 3 days of delivery in addition to the 6 week visit specified within guidelines from 2008 and prior. This change may have enhanced health messaging focused on the postnatal period to influence an increase in attendance to early PNC.

Our results highlighted the need to reinforce messages about the importance of completing all health behavior and health visit recommendations along the continuum of care, regardless of infant HIV exposure status. Lessons should be taken from provinces achieving higher uptake of PNC (such as Free State Province and North West Province) to improve uptake in lower performing provinces (such as Gauteng Province and Eastern Cape Province). Messages about the importance of PNC should target those who do not attend at least four ANC, those who initiate ANC after 12 weeks gestation, those who deliver outside of the clinic setting, and those with demographic characteristics predictive of lower PNC utilization.

### Limitations

As a secondary data analysis, the original survey was not designed to specifically answer the question addressed by this analysis. By the nature of this study design, responses to the questions about health behavior were retroactive and may have introduced information bias; however there is no reason to believe that maternal memory was affected differentially between the group who met the recommended number of PNC visits and those who did not. Infants that died before 4–8 weeks and those who were severely ill were excluded from the study based on the survey sampling method, even though their cases could have been some of the most informative regarding the importance of PNC.

Only the responses of mothers were included in this study (excluding responses from other caregivers (3.2% of survey participants). Mothers were excluded from this analysis if they did not provide information about number of PNC visits, however the proportion missing PNC responses was only 6.93% of mothers. . The potential effects of missing data were accounted for in the process of developing weights to analyze this data set as representative of the national population of mothers in South Africa. For some variables, the proportion of “Do not know” or “No answer” responses was greater than 10%, thus interpretability is limited. We did not ascertain information regarding the timing, contents, and quality of PNC visits, thus limiting the extent to which we could assess the productiveness of the care received. The location where ANC, delivery care, and PNC were received was not collected within this study, thus our ability to explain patterns in PNC uptake by district were limited to province of residence. This study focused on health care received in the formal health system and did not include information about home care or traditional care.

## CONCLUSIONS

While South Africa has not yet officially adopted the WHO recommendation for 3 early PNC visits as the national standard of practice, uptake of these important visits is high and coverage appears to have increased between 2010 and 2013. Uptake of at least 2 PNC visits, the current national standard in South Africa, is very high (over 94%), indicating strong implementation of this guideline—an encouraging prospect for potential implementation of the WHO recommendation for 3 early PNC visits. South Africa seen only mild improvements in infant survival over the past decade and has not seen improvements in neonatal survival. Adoption of the global postnatal guideline for 3 early PNC visits, or adoption of more community–based postnatal care, may be the next necessary step toward achieving optimal neonatal and infant health outcomes. Our findings suggest that efforts to encourage a woman’s compliance with global recommendations along the continuum of care–such as facility–based delivery, timely initiation of ANC, and achieving 4 or more ANC visits–increase the likelihood of her infant completing the recommended number of early PNC. Messages from the health system should be strengthened during ANC, labor and delivery, and PNC to ensure that all expectant and breastfeeding mothers understand the minimum recommended visits along the continuum of care and work with health care providers to achieve them. These messages should specifically target women who have been non–adherent to at least one recommended health behavior or interaction with a facility.

As HIV prevention moves toward elimination of maternal to child HIV transmission and reduction in neonatal mortality, South Africa must strengthen the implementation of policies and programs aimed at achieving high utilization of early PNC in order to ensure the health and well–being of new generations during the postnatal period and beyond.

## References

[1] You D, Wardlaw T, Salama P, Jones G (2010). Levels and trends in under-5 mortality, 1990-2008.. Lancet.

[2] Sartorius B, Sartorius K (2014). Global infant mortality trends and attributable determinants – an ecological study using data from 192 countries for the period 1990–2011.. Popul Health Metr.

[3] World Health Organization. Every Newborn: an action plan to end preventable deaths. 2014. Available: http://apps.who.int/iris/handle/10665/127938. Accessed: 9 February 2016.

[4] World Health Organization. Postnatal care of the mother and newborn 2013. 2013. Available: http://apps.who.int/iris/bitstream/10665/97603/1/9789241506649_eng.pdf. Accessed: 8 October 2015. 24624481

[5] World Health Organization. PMTCT strategic vision 2010-2015. World Health Organization: Geneva: 2010.

[6] Baleta A (2011). South Africa takes steps to reduce perinatal mortality.. Lancet.

[7] Dorrington R, Bradshaw D, Laubscher R, Nannan N. Rapid mortality surveillance report 2013. 2014. Available: http://www.mrc.ac.za/bod/RapidMortalitySurveillanceReport2014.pdf. Accessed: 8 October 2015.

[8] Health Systems Trust. The 2013 National Antenatal Sentinel HIV Prevalence Survey South Africa. 2014. Available: http://www.hst.org.za/publications/2013-national-antenatal-sentinel-hiv-prevalence-survey-south-africa. Accessed: 13 December 2015.

[9] Newell M-L, Coovadia H, Cortina-Borja M, Rollins N, Gaillard P, Dabis F (2004). Mortality of infected and uninfected infants born to HIV-infected mothers in Africa: a pooled analysis.. Lancet.

[10] Goga A, Dinh T-H, Jackson D. Evaluation of the effectiveness of the national Prevention of Mother-to-Child Transmission (PMTCT) programme on infant HIV measured at six weeks postpartum in South Africa. South African Medical Research Council, National Department of Health South Africa and PEPFAR/US Centers for Disease Control & Prevention. 2012. Available: http://repository.uwc.ac.za/xmlui/handle/10566/462. Accessed: 9 February 2016.

[11] Fadnes LT, Jackson D, Engebretsen IMS, Zembe W, Sanders D, Sommerfelt H (2011). Vaccination coverage and timeliness in three South African areas: a prospective study.. BMC Public Health.

[12] Bursac Z, Gauss CH, Williams DK, Hosmer DW (2008). Purposeful selection of variables in logistic regression.. Source Code Biol Med.

[13] Sines BE, Syed U, Wall S, Worley H. Postnatal Care: A Critical Opportunity to save mothers and newborns. 2006. Available: http://www.prb.org/pdf07/snl_pncbrieffinal.pdf. Accessed: 8 October 2015.

[14] Massy N, Peer N, English R, Padarath A, Barron P, Day C, editors. District Health Barometer 2015/16. Durban: Health Systems Trust; 2016.

[15] Goga A, Jackson D, Singh M, Lombard C. Group S study. 2012-2013 SAPMTCTE Report: Early (4-8 weeks postpartum) Population-level Effectiveness of WHO PMTCT Option A, South Africa. Available: http://www.mrc.ac.za/healthsystems/SAPMTCTEReport2012.pdf. Accessed: 5 February 2016.

[16] Bendel RB, Afifi AA (1977). Comparison of stopping rules in forward “Stepwise” regression.. J Am Stat Assoc.

[17] Mickey RM, Greendland S (1989). The impact of confounder selection criteria on effect estimation.. Am J Epidemiol.

[18] The World Bank Group. Country Income Groups (World Bank Classification), Country and Lending Groups. 2011. Available: http://data.worldbank.org/about/country-classifications/country-and-lending-groups. Accessed: 4 March 2016.

[19] Kanté AM, Chung CE, Larsen AM, Exavery A, Tani K, Phillips JF (2015). Factors associated with compliance with the recommended frequency of postnatal care services in three rural districts of Tanzania.. BMC Pregnancy Childbirth.

[20] Yeji F, Shibanuma A, Oduro A, Debpuur C, Kikuchi K, Owusu-Agei S (2015). Continuum of care in a maternal, newborn and child health program in Ghana: low completion rate and multiple obstacle factors.. PLoS One.

[21] Somefun OD, Ibisomi L (2016). Determinants of postnatal care non-utilization among women in Nigeria.. BMC Res Notes.

[22] Titaley CR, Hunter CL, Heywood P, Dibley MJ (2010). Why don’t some women attend antenatal and postnatal care services?: a qualitative study of community members’ perspectives in Garut, Sukabumi and Ciamis districts of West Java Province, Indonesia.. BMC Pregnancy Childbirth.

[23] De Schacht C, Lucas C, Mboa C, Gill M, Macasse E, Dimande SA (2014). Access to HIV prevention and care for HIV-exposed and HIV-infected children: a qualitative study in rural and urban Mozambique.. BMC Public Health.

[24] Mohan D, Gupta S, LeFevre A, Bazant E, Killewo J, Baqui AH (2015). Determinants of postnatal care use at health facilities in rural Tanzania: multilevel analysis of a household survey.. BMC Pregnancy Childbirth.

[25] Nankwanga A. Factors influencing utilisation of postnatal services in Mulago and Mengo Hospitals Kampala, Uganda. University of the Western Cape. Available: http://etd.uwc.ac.za/xmlui/handle/11394/237. Accessed: 27 September 2015.

[26] Tlebere P, Jackson D, Loveday M, Matizirofa L, Mbombo N, Doherty T (2007). Community-based situation analysis of maternal and neonatal care in South Africa to explore factors that impact utilization of maternal health services.. J Midwifery Womens Health.

[27] Landau LB. Gauteng 2055 Trend Paper: Population & Migration. 2008; Available: http://migration.org.za. Accessed: 9 February 2016.

[28] South African Census Bureau. Statistical release (Revised) Census 2011. 2012;78. Available: www.statssa.gov.za. Accessed: 9 February 2016.

[29] Gushulak BD, MacPherson DW (2006). The basic principles of migration health: population mobility and gaps in disease prevalence.. Emerg Themes Epidemiol.

[30] Bhardwaj S, Barron P, Pillay Y, Treger-Slavin L, Robinson P, Goga A (2014). Elimination of mother-to-child transmission of HIV in South Africa: Rapid scale-up using quality improvement.. S Afr Med J.

[31] National Department of Health of South Africa. Guidelines for Maternity Care in South Africa. Pretoria, South Africa; 2007.

[32] National Department of Health of South Africa. Policy and Guidelines for the Implementation of the PMTC Programme. National Department of Health; Pretoria: 2008.

[33] The National Department of Health. National Consolidated Guidelines for the Prevention of Mother-To-Child Transmission of HIV (PMTCT) and the Management of HIV in Children, Adolescents and Adults. National Department of Health; Pretoria; 2015.

[34] National Department of Health of South Africa. The South African Antiretroviral Treatment Guidelines 2013. 2013. Available: http://www.sahivsoc.org/upload/documents/2013 ART Guidelines-Short Combined FINAL draft guidelines 14 March 2013.pdf. Accessed: 25 March 2016.

[35] National Department of Health of South Africa. The South African Antiretroviral Treatment Guidelines. 2010; Available: http://apps.who.int/medicinedocs/documents/s19153en/s19153en.pdf. Accessed: 25 March 2016.

[36] Goga AE, Doherty T, Jackson DJ, Sanders D, Colvin M, Chopra M (2012). Infant feeding practices at routine PMTCT sites, South Africa: results of a prospective observational study amongst HIV exposed and unexposed infants - birth to 9 months.. Int Breastfeed J.

[37] World Health Organization. Pregnancy, childbirth, postpartum and newborn care: a guideline for essential practice. World Health Organization: Geneva; 2006. 26561684

[38] de Wet K, Wouters E, Engelbrecht M (2011). Exploring task-shifting practices in antiretroviral treatment facilities in the Free State Province, South Africa.. J Public Health Policy.

[39] National Department of Health of South Africa. National Department of Health Strategic Plan 2010/11-2012/13. Department of Health; Pretoria; 2014.

[40] le Roux IM, Tomlinson M, Harwood JM, O’Conner MJ, Worthman CM, Mbewu N (2013). Outcomes of home visits for pregnant mothers and their infants: a cluster randomised controlled trial.. AIDS.

[41] Adam MB, Dillmann M, Chen M, Mbugua S, Ndung’u J, Mumbi P (2014). Improving maternal and newborn health: effectiveness of a community health worker program in rural Kenya.. PLoS One.

[42] Beauclair R, Petro G, Myer L (2014). The association between timing of initiation of antenatal care and stillbirths: a retrospective cohort study of pregnant women in Cape Town, South Africa.. BMC Pregnancy Childbirth.

[43] Nabukera SK, Witte K, Muchunguzi C, Bajunirwe F, Batwala VK, Mulogo EM (2006). Use of postpartum health services in rural Uganda: knowledge, attitudes, and barriers.. J Community Health.

[44] Mpembeni RN, Killewo JZ, Leshabari MT, Massawe SN, Jahn A, Mushi D (2007). Use pattern of maternal health services and determinants of skilled care during delivery in Southern Tanzania: implications for achievement of MDG-5 targets.. BMC Pregnancy Childbirth.

[45] Ferrer R, Klein WM (2015). Risk perceptions and health behavior.. Curr Opin Psychol..

[46] Becker M (1974). The Health Belief Model and Sick Role Behavior.. Health Educ Behav.

[47] Brewer NT, Chapman GB, Gibbons FX, Gerrard M, McCaul KD, Weinstein ND (2007). Meta-analysis of the relationship between risk perception and health behavior: The example of vaccination.. Health Psychol.

[48] Burgard SA, Treiman DJ (2006). Trends and racial differences in infant mortality in South Africa.. Soc Sci Med.

[49] South Africa Demographic and Health Survey 2003. Available: https://dhsprogram.com/pubs/pdf/FR206/FR206.pdf. Accessed: 28 September 2015.

[50] Dhakal S, Chapman GN, Simkhada PP, van Teijlingen ER, Stephens J, Raja AE (2007). Utilisation of postnatal care among rural women in Nepal.. BMC Pregnancy Childbirth.

[51] Magadi MA, Madise NJ, Rodrigues RN (2000). Frequency and timing of antenatal care in Kenya: Explaining the variations between women of different communities.. Soc Sci Med.

